# Bilateral Femur and Humerus Avascular Necrosis Associated With Corticosteroids: A Rare Case Presentation

**DOI:** 10.7759/cureus.50834

**Published:** 2023-12-20

**Authors:** Mert Can Çavuş, Muhammed Yusuf Afacan, Arda Zeytunlu, Aliekber Yapar

**Affiliations:** 1 Department of Orthopaedics and Traumatology, Istanbul University-Cerrahpasa, Cerrahpasa Faculty of Medicine, Istanbul, TUR; 2 General Practice, Antalya Kepez District Health Department, Antalya, TUR; 3 Department of Orthopaedics and Traumatology, University of Health Sciences, Antalya Training and Research Hospital, Antalya, TUR

**Keywords:** core decompression, covid-19, bilateral femoral heads, bilateral humeral heads, multifocal involvement, corticosteroid-associated avascular necrosis, osteonecrosis, avascular necrosis of the humeral head, avascular necrosis of the femoral head

## Abstract

Avascular necrosis of the bone is a pathology characterized by compromised blood circulation, leading to necrosis due to insufficient vascular nourishment. Within the realm of orthopedics and traumatology, instances of avascular necrosis are steadily increasing. Notably, the escalating use of corticosteroids in managing inflammatory diseases and acute respiratory distress syndrome associated with the COVID-19 pandemic has resulted in a surge of outpatient referrals concerning cases of glucocorticoid-associated avascular necrosis. This study aims to elucidate the management of avascular necrosis following oral corticosteroid use in a young and otherwise healthy male patient, impacting both humeral and femoral heads bilaterally.

A 26-year-old adult male, devoid of chronic health conditions, received a diagnosis of bilateral avascular necrosis in humeral and femoral heads within two years following a one-month course of oral corticosteroids. The patient underwent a comprehensive treatment regimen, encompassing hyperbaric oxygen therapy, oral antiplatelet therapy, a tailored physical therapy and rehabilitation program, and bilateral core decompression surgery for both hip joints. During the three-year follow-up, the patient exhibited a favorable response to treatment, demonstrating a complete and painless range of motion in both shoulder and hip joints. This case serves to underscore a crucial point: femoral head avascular necrosis may not invariably manifest as the initial bone affected, and a substantial time lapse may transpire between corticosteroid use and the onset of clinical symptoms. We emphasize the critical importance of not dismissing complaints pertaining to other bones in patients with a confirmed diagnosis and stress the significance of prompt detection in avascular necrosis. Furthermore, this study highlights the necessity for heightened vigilance in instances of orthopedic grievances among individuals with a history of corticosteroid use, particularly those related to the pandemic and inflammatory diseases, to facilitate early diagnosis and intervention for avascular necrosis.

## Introduction

During the COVID-19 pandemic, while the focus was primarily on pandemic-related mortality, post-pandemic attention is increasingly directed toward pandemic-related morbidities. As orthopedic and trauma specialists, we routinely encounter the enduring repercussions of corticosteroid applications administered during the treatment of acute respiratory distress syndrome throughout the pandemic [1].

Avascular necrosis, also recognized as osteonecrosis, ischemic necrosis, and aseptic necrosis, can result from various factors, including trauma, glucocorticoid use, excessive alcohol consumption, sickle cell anemia, hereditary coagulopathies, radiotherapy, chemotherapy, Gaucher's disease, and Caisson disease. Certain viruses, such as HIV, pancreatitis, and osteomyelitis, have also been correlated with osteonecrosis [2]. While the etiology of avascular necrosis remains incompletely elucidated in the literature, one well-established causal factor is the use of corticosteroids [3,4]. Corticosteroid-associated avascular necrosis predominantly impacts the femoral head in 75% of cases [4]. Although the research primarily focuses on this aspect, other documented cases involve the proximal humerus, knee, and wrist [3].

In this particular case, we aim to illustrate that corticosteroid-associated avascular necrosis may not manifest promptly after corticosteroid use and can encompass all four major bones in a patient. Additionally, through this unique case, we intend to underscore the significance of addressing bone and joint complaints beyond the directly affected area in avascular necrosis cases, a frequency observed to be on the rise in the post-pandemic period.

## Case presentation

**Figure 1:**
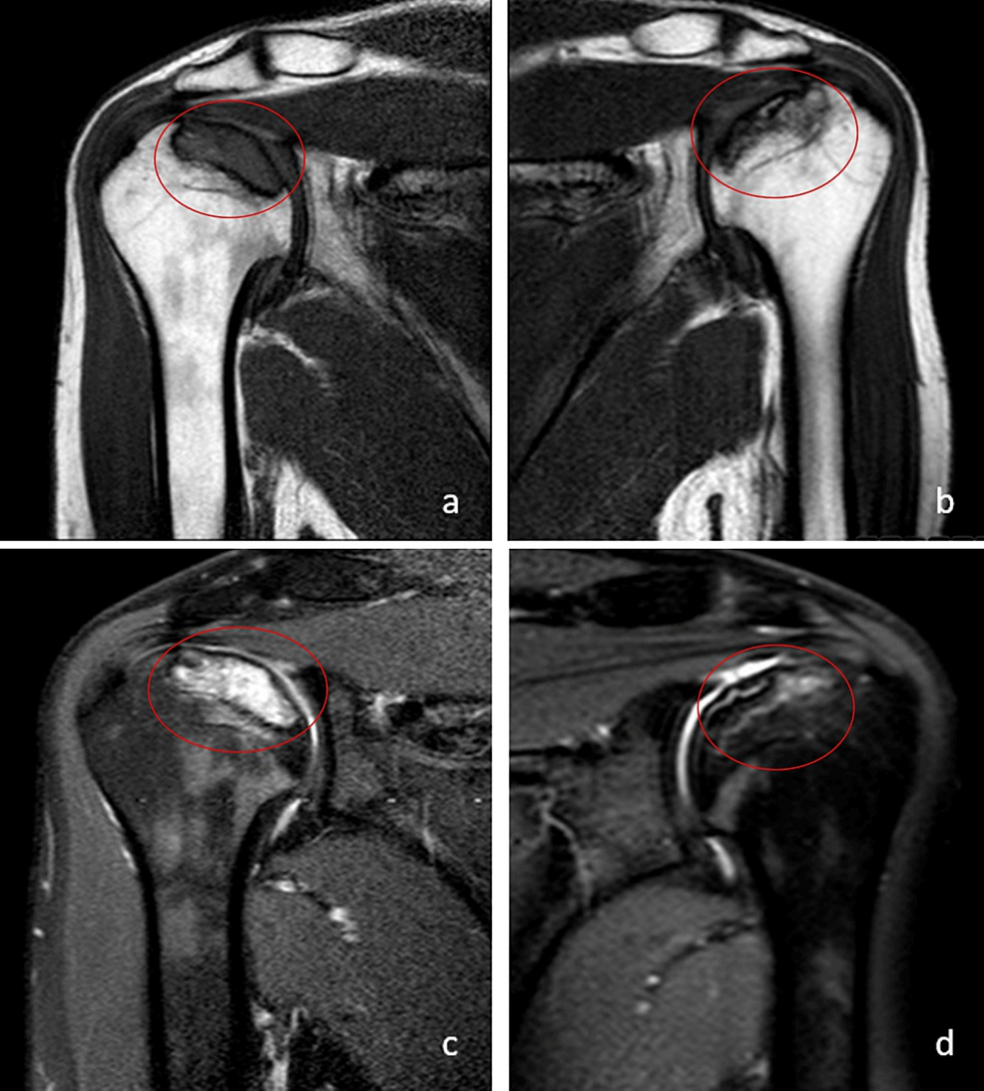
T1 and T2 sequence magnetic resonance images depicting avascular necrosis involvement of bilateral humeral heads. T1 sequence in the proximal right humerus (a) and in the proximal left humerus (b); T2 sequence in the proximal right humerus (c) and in the proximal left humerus (d). The lesions are marked with a red ellipse.

A 26-year-old male patient received a daily dosage of 48 mg oral methylprednisolone for one month in July 2018, as part of preoperative preparation for nasal polyp surgery administered by an ear, nose, and throat specialist. Following this, a controlled dose reduction was implemented over a span of 10 days. The patient sought consultation at the orthopedics and traumatology outpatient clinic in December 2020, reporting pain during right shoulder flexion that had commenced in October 2020. There was no evidence of trauma, alcohol or substance abuse, relevant medical conditions (such as lupus, sickle cell disease, HIV, or coagulation disorders), history of radiation exposure, thrombotic disorders, hormonal imbalances, or vascular disorders in the patient's medical history. Upon examination, painful flexion exceeding 90 degrees in the right shoulder, coupled with positive Neer, Hawkins, and Jobe tests, led to considerations of rotator cuff pathologies and subacromial impingement. Direct radiography and contrast-free shoulder MRI were conducted, revealing Stage 1 avascular necrosis in the proximal right humerus (Figures 1a, 1c). The prescribed management included a shoulder-arm sling and the initiation of a physical therapy and rehabilitation program. Nine months later, analogous complaints surfaced in the left shoulder, prompting a contrast-free shoulder MRI, which disclosed newly onset avascular necrosis findings in the proximal left humerus (Figures 1b, 1d).

**Figure 2:**
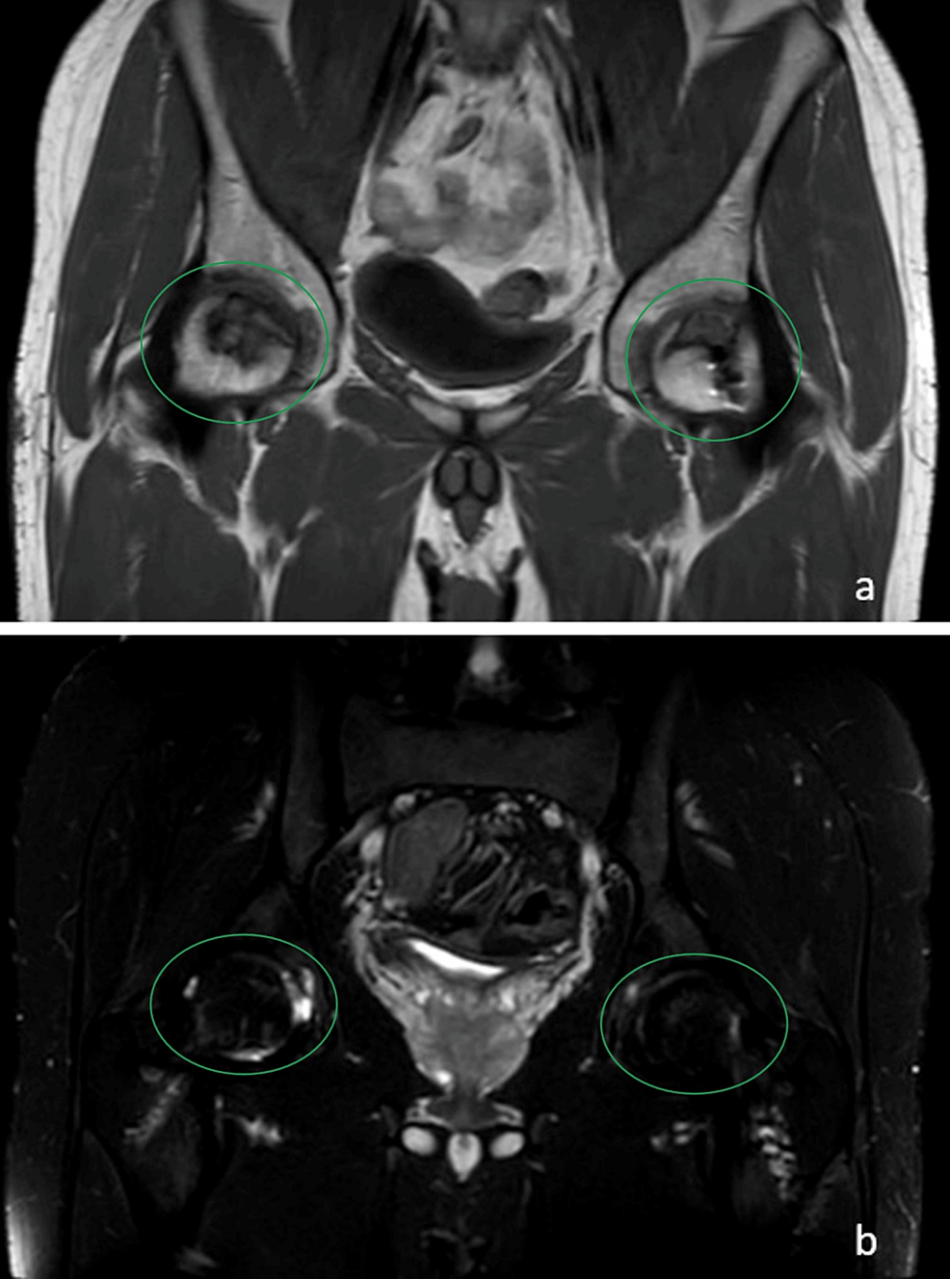
Bilateral hip magnetic resonance images of the patient. T1 (a) and T2 (b) sequence magnetic resonance images illustrating avascular necrosis involvement of bilateral femoral heads. The lesions are marked with green ellipses.

The patient underwent 45 sessions of hyperbaric oxygen therapy. Subsequently, mild discomfort in the right hip during prolonged walks emerged, leading to a thorough examination that unveiled complete yet painful joint movements. A bilateral hip MRI displayed early-stage avascular necrosis of the femoral head, particularly evident in the left hip (Figures 2a, 2b). 

Bilateral core decompression surgery was performed in December 2022. Post-surgery, hyperbaric oxygen therapy, along with the physical therapy and rehabilitation program, was sustained. Additionally, the patient commenced a daily regimen of acetylsalicylic acid at 100 mg. Presently, the patient exhibits a full and painless range of motion in both hip joints. Hyperbaric oxygen therapy, coupled with concurrent physical therapy and rehabilitation, contributed positively to the patient's recovery. Early diagnosis and timely conservative treatment obviated the necessity for surgical intervention in bilateral proximal humerus avascular necrosis. In the three-year follow-up, the patient maintains a full and painless range of motion in both shoulder and hip joints.

## Discussion

While the underlying pathology of avascular necrosis remains not fully understood, a spectrum of factors, including vascular damage, mechanical stress, oxidative stress, adipocyte dysfunction, apoptosis defects, and coagulation disorders, can manifest in isolation or combination, ultimately leading to compromised blood supply in the watershed areas of bone tissue. Among non-traumatic cases, glucocorticoid-associated avascular necrosis stands out as the most prevalent cause [2]. The sensitivity to corticosteroid-associated avascular necrosis is believed to hinge on diverse factors, encompassing genetic predisposition, the duration and quantity of glucocorticoid exposure, and underlying medical conditions [5]. In a prospective study involving 337 patients, Shigemura et al. reported a heightened frequency of corticosteroid-associated avascular necrosis in systemic lupus erythematosus patients compared to individuals with other collagen diseases [6].

Although femoral head involvement constitutes 75% of corticosteroid-associated avascular necrosis cases, the proximal humerus follows as the second most affected region. However, multifocal involvement is reported in only 3% of cases within a study involving 1056 avascular necrosis patients [7]. Furthermore, the occurrence of multifocal involvement affecting both femoral and humeral heads is an exceptionally rare condition, making our case notably striking. The correlation between corticosteroid-associated avascular necrosis and the dose and duration of corticosteroid use is well-established. A retrospective study involving 113,734 patients who used corticosteroids revealed that those diagnosed with corticosteroid-associated avascular necrosis received the diagnosis, on average, 219 (± 374) days after corticosteroid use, with an average history of 3314 mg (± 2908) prednisolone or equivalent corticosteroid use [8]. In our case, the patient utilized a 1950-milligram equivalent dose of methylprednisolone, and the initial diagnosis was made approximately 780 days after corticosteroid use.

According to the 2017 Delphi survey conducted by the Association Research Circulation Osseous (ARCO) with 28 experts worldwide on osteonecrosis, corticosteroid-associated avascular necrosis was classified by consensus with the following criteria: The patient should have a history of using more than 2 grams of prednisolone or equivalent glucocorticoid within three months to classify a case as corticosteroid-associated avascular necrosis. The diagnosis should be made within two years following the use of this dose of glucocorticoid. There should be no other risk factors explaining osteonecrosis. Another key outcome of the survey is that despite studies suggesting that some diseases treated with glucocorticoids predispose to corticosteroid-associated avascular necrosis, the consensus emphasizes that the criteria established should be evaluated independently of the underlying disease [5]. In our study, comprehensive assessment revealed no evidence of osteonecrosis risk factors, including the presence of trauma, alcohol or substance abuse, relevant medical conditions (such as lupus, sickle cell disease, HIV, or coagulation disorders), history of radiation exposure, thrombotic disorders, hormonal imbalances, or vascular disorders in the patient's medical history. The only risk factor was approximately 2 gram use of the corticosteroids.

The noteworthy aspect of our case lies in the proximal humerus being the first affected area, underscoring the importance of comprehensive medical history and questioning regarding comorbidities and corticosteroid use in the diagnosis of avascular necrosis. Particularly, in the early stages where findings may not be apparent on X-rays, avascular necrosis is a diagnosis that can be overlooked, especially in patients presenting with shoulder pain in outpatient clinics. Additionally, it is crucial to assess new complaints accompanying or emerging in follow-ups of patients diagnosed with avascular necrosis for possible multifocal involvement. The escalated use of corticosteroids during the COVID-19 pandemic in the treatment protocols of acute respiratory distress syndrome has led to an increased incidence of corticosteroid-associated adverse effects in the community [1]. Hence, we advocate considering this situation during history-taking and clinical examination in the clinic.

## Conclusions

This case serves to underscore several critical points. Firstly, it highlights that femoral head avascular necrosis, often emphasized in the literature, may not manifest as the initial affected bone. Clinical symptoms may not promptly emerge after corticosteroid use, and multiple bones can be affected at different times during this process. Importantly, complaints in other bones of diagnosed patients should not be overlooked.

Furthermore, we emphasize that in the early stages of avascular necrosis, conservative treatment coupled with timely rehabilitation may prove sufficient for the humeral head, which is not load-bearing. On the other hand, for the load-bearing femoral head, achieving a long-term complete and painless joint range of motion is attainable through core decompression.

This rare case, involving bilateral humerus and subsequent femoral head involvement, serves as a poignant illustration of the imperative need for close monitoring for avascular necrosis in patients with corticosteroid use, particularly in the context of factors such as the ongoing pandemic.
